# Foreign Body Ingestion Ending Up in Late-Presenting Morgagni Hernia Diagnosis: A Case Report

**DOI:** 10.7759/cureus.63754

**Published:** 2024-07-03

**Authors:** Elisavet Kanna, Zoi Lamprinou, Ioannis Skondras, Adelais Tzortzopoulou, Orthodoxos Achilleos

**Affiliations:** 1 2nd Pediatric Surgery Department, Panagiotis & Aglaia Kyriakou Childrens' Hospital, Athens, GRC

**Keywords:** foreign body ingestion treatment, congenital diaphragmatic hernia, children, late presentation, laparoscopic repair, thoracoscopic surgery

## Abstract

Morgagni hernia (MH), also known as a retrosternal or parasternal hernia, is a rare type of congenital diaphragmatic hernia (CDH) characterized by a defect in the anterior diaphragm. Patients with late-diagnosed MH typically present with vague gastrointestinal or respiratory symptoms. In some instances, MH is incidentally identified through chest X-rays performed for other reasons, such as foreign body ingestion, as illustrated in our presented case. We present a case of a delayed congenital diaphragmatic hernia of the Morgagni type in a two-year-old boy with a history of foreign body ingestion and severe abdominal pain. Diagnostic imaging, including chest radiograph and computed tomography (CT) scan, confirmed the diaphragmatic defect. Surgical repair, performed laparoscopically, resulted in an uncomplicated postoperative course and a favorable long-term outcome.

## Introduction

Morgagni hernia (MH), named after the Italian anatomist Giovanni Battista Morgagni, is a rare type of congenital diaphragmatic hernia characterized by an anterior and retrosternal defect in the diaphragm, that appears when the fibrotendinous tissue originating from the costochondral arches fails to fuse with the fibrotendinous part of the pars sternalis. This type of hernia was first described by Morgagni in 1769, represents 2% of congenital diaphragmatic hernia (CDH) and the diagnosis can be delayed due to its rarity as well as its ambiguous and nonspecific presentation [1]. The majority of MH cases are asymptomatic and discovered by accident, even late in adulthood.

The subset of patients with late diagnosis usually present with vague gastrointestinal or respiratory symptoms. In some cases, they may also be incidentally diagnosed by chest X-rays done for other causes, such as foreign body ingestion which is described below for our patient [2].

We present a case of a late-presenting congenital MH and our purpose is both to point out the diagnostic challenges associated with this condition and highlight the role of imaging modalities such as chest radiography and computed tomography scans in achieving an accurate diagnosis. We also explore the surgical management alternatives for MH, including minimally invasive techniques such as laparoscopy, which offer favourable outcomes for patients with diaphragmatic defects like MH.

## Case presentation

A two-year-old male toddler presented with a medical history of recent ingestion of a foreign body. Upon arrival at the emergency department, the patient had intense borborygmus, indicating increased bowel sounds, and severe intermittent epigastric discomfort but nevertheless without respiratory distress. The vital signs were within range for the patient’s age and the chest auscultation revealed slightly decreased breath sounds in the lower right lung field. The patient's symptoms and medical history raised concerns for possible complications and a chest radiograph was promptly ordered to investigate the underlying cause.

**Figure 1:**
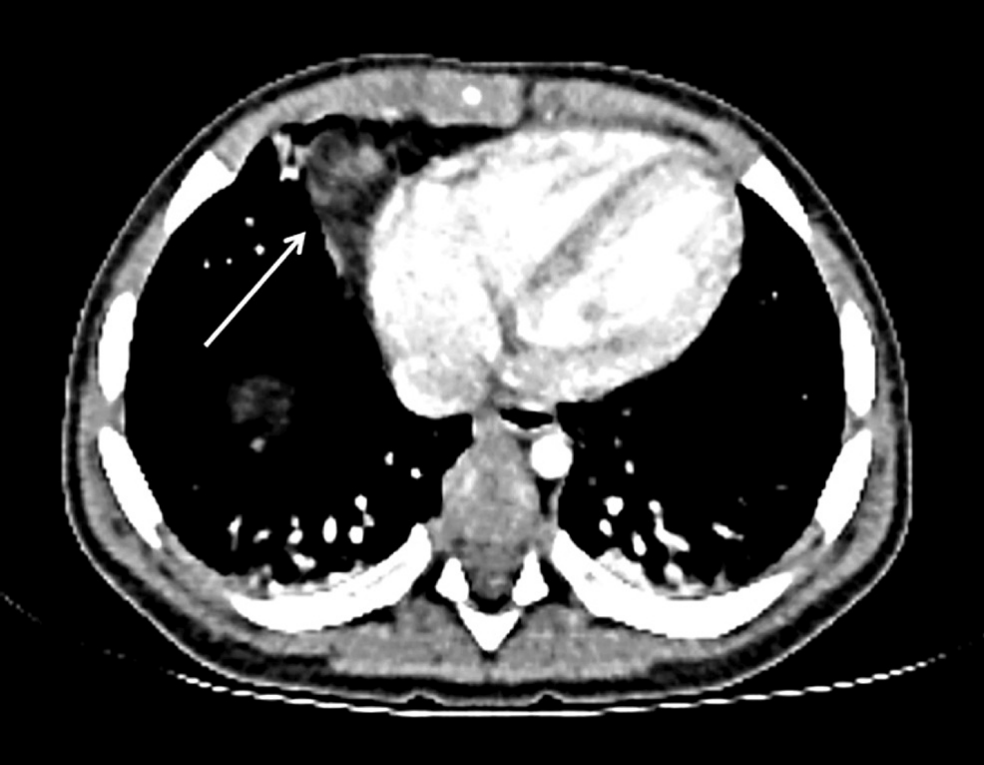
Contrast-enhanced computed tomography showing herniated omentum into the right hemithorax (axial view).

The chest radiograph did not reveal the expected foreign body but, to our surprise, an unpredictable finding came up; loops of small bowel and omentum occupied the right hemithorax, suggestive of a diaphragmatic hernia. Subsequent confirmation of the diaphragmatic defect was obtained through a computed tomography scan, further highlighting the urgency of addressing the hernia (Figure 1).

**Figure 2:**
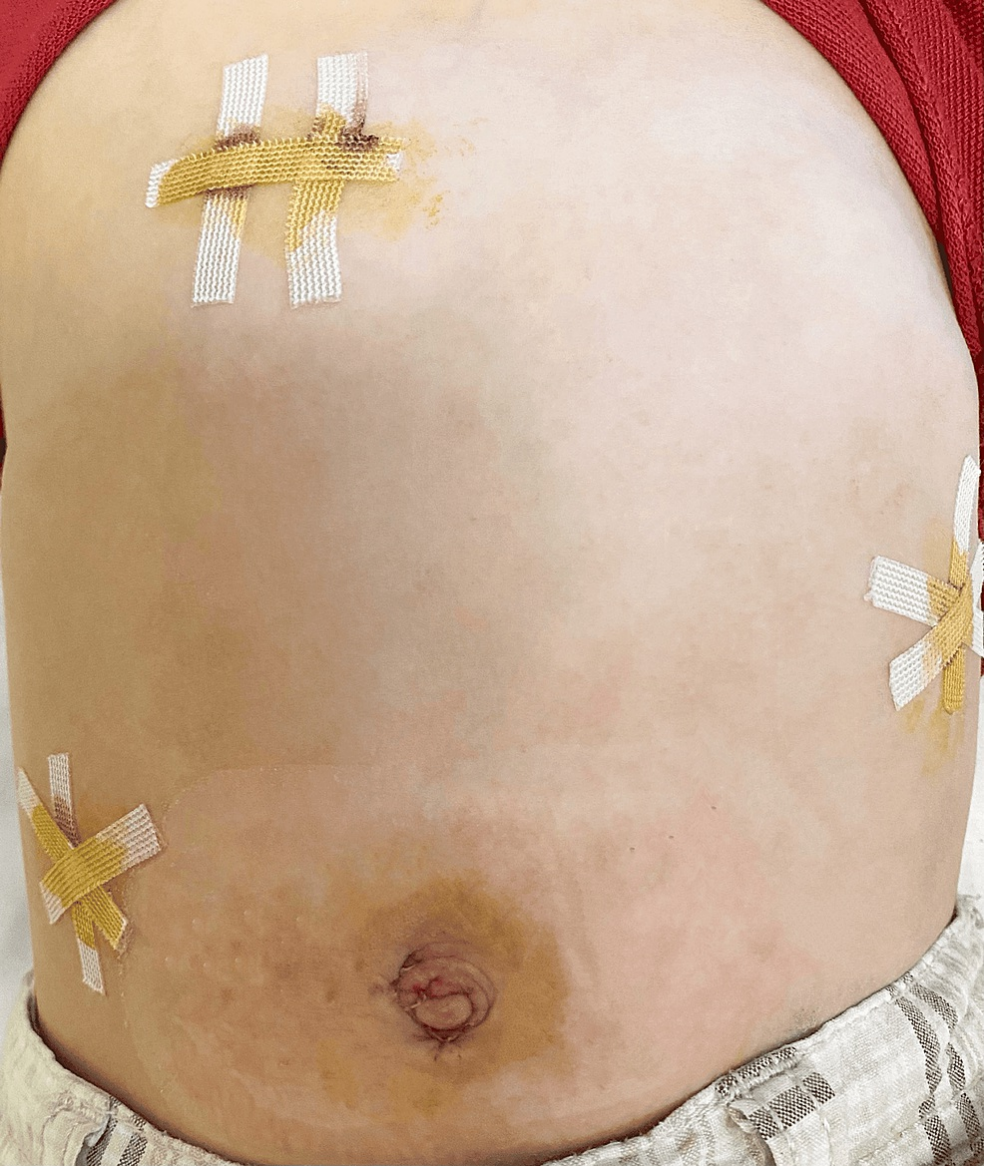
Final photograph after laparoscopic repair of congenital diaphragmatic hernia (CDH) on the third postoperative day.

Considering the patient's age and the nature of the diaphragmatic defect, a decision was made to pursue laparoscopic repair as the most appropriate treatment option. Under general anesthesia, a laparoscopic operation was performed, with the insertion of a 5mm port into the umbilical region and three 3mm ports at strategic locations as shown in the picture below (Figure 2).

**Figure 3:**
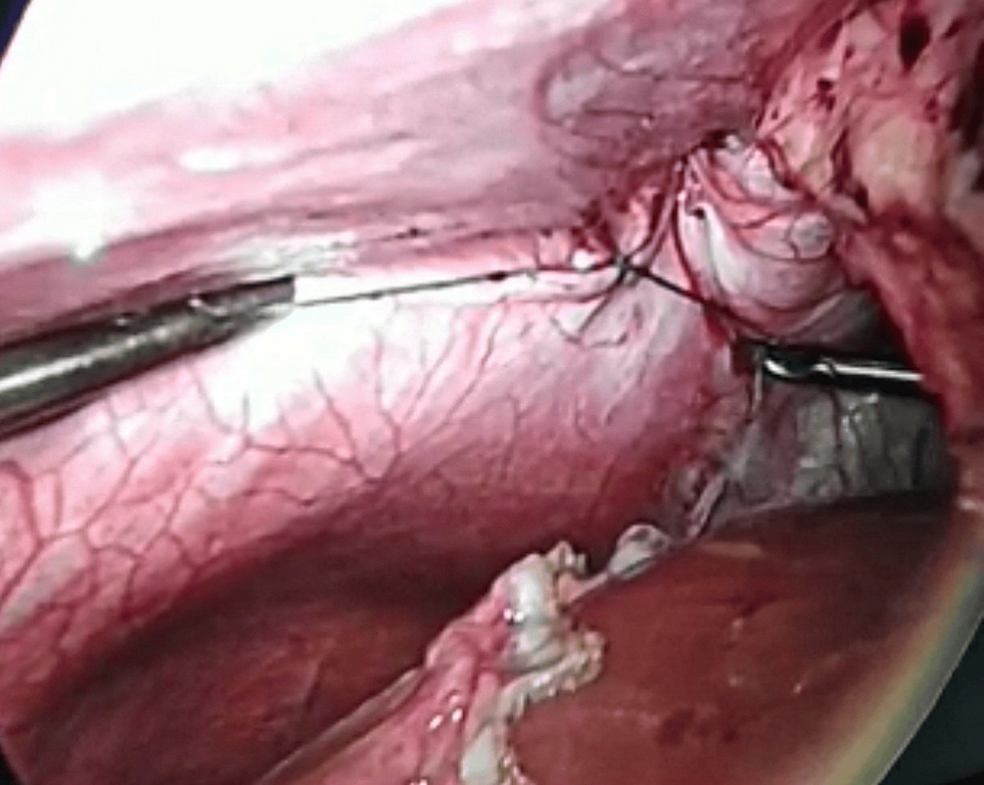
The appearance of a closed diaphragmatic orifice with intracorporeal interrupted non-absorbable sutures.

During the surgery, the presence of a right-sided hernia protruding from the anterior and retrosternal aspect of the diaphragm was immediately apparent. A punctilious dissection was carried out, and a small hernia sac was excised to expose the defect, reducing the risk of recurrence and ensuring a more durable repair. The defect was then meticulously repaired using intracorporeal interrupted non-absorbable sutures (Ethibond 2/0 - 17mm), assuring a secure closure (Figure 3).

Following the laparoscopic repair, the patient's postoperative course was uneventful, with no complications observed. He was discharged from the hospital on the third postoperative day, demonstrating satisfactory recovery. Subsequent follow-up evaluations over a two-year period revealed no recurrence of symptoms, indicating successful resolution of the diaphragmatic hernia.

## Discussion

Morgagni hernia, also known as a retrosternal or parasternal hernia, is a rare type of congenital diaphragmatic hernia characterized by a defect in the anterior diaphragm and account for approximately 2-3% of all diaphragmatic hernias. They typically manifest in childhood or adolescence, however, they can also remain undetected until maturity on occasion. They are less common than posterolateral (Bochdalek) hernias, which account for the majority of CDH cases and are detected either before birth or shortly after delivery and are typically addressed within the first month of life [1,3].

They are frequently associated with other congenital anomalies, reflecting their developmental origins. These associated conditions can influence the clinical management and prognosis. Common anomalies include: cardiovascular abnormalities, particularly atrial septal defects and tetralogy of Fallot, genetic syndrome (especially Down syndrome), malrotation and chest wall deformities [4]. In the pediatric age group, MH is rare, accounting for about 1-5% of all types of congenital diaphragmatic hernia [2, 4, 5]. Despite the potential for associated anomalies, children with MH often exhibit milder symptoms, leading to delayed diagnosis [1]. Patients may exhibit a complete lack of symptoms or rarely may present with chronic, acute, or sudden manifestations. Some symptoms include dyspnea, especially during physical activity, chest pain, recurrent infections in the respiratory tract, or gastrointestinal issues such as nausea or abdominal pain. It is noteworthy that none of the symptoms seen in older infants with MH may be specifically linked to this defect, in contrast to newborn posterolateral hernias, which usually result in respiratory distress directly after birth [5].

Late-presenting MH is generally considered a benign condition with a favorable prognosis [6]. Unlike posterolateral defects, which can be difficult to visualize laparoscopically, anterior defects are more readily identified using this approach.

The initial diagnostic tool for late-presenting MH is typically a chest radiograph, which can identify the majority of cases. However, it's crucial to recognize that radiographs may occasionally yield misinterpretations, resembling conditions such as lower lobe pneumonia, pneumothorax, pleural effusion, diaphragmatic eventration, or congenital cystic adenomatoid malformation, thereby potentially leading to diagnostic errors. Consequently, such misdiagnoses, coupled with nonspecific clinical presentations, might delay accurate identification. The diagnosis is confirmed by performing additional imaging modalities like chest or abdominal CT scans or magnetic resonance imaging (MRI) [7, 8]. These specify the defect's dimensions and the hernial sac's contents.

Treatment for a late presenting MH typically involves surgical repair of the hernia to prevent further organ herniation and to restore normal respiratory function. The timing of surgery will depend on the severity of the symptoms and the presence of other associated conditions. In some cases, the hernia may be repaired with minimally invasive techniques, while in other cases, open surgery may be necessary.

The decision to excise or leave the hernia sac during repair remains a topic of debate and controversy in the medical community. Traditionally, in cases treated using the open surgical approach, the hernia sac is typically excised. This approach aims to remove the sac completely to prevent potential complications such as recurrence or incarceration of abdominal contents [9]. However, with the advent of laparoscopic-assisted techniques for CDH repair, the management of the hernia sac has become less standardized. In some laparoscopic-assisted repairs, the choice is to leave the hernia sac intact, particularly if it is small and handily reducible to preserve the integrity of surrounding tissues and minimize the risk of injury to vital structures. The decision to excise or leave the hernia sac during laparoscopic repair may depend on various factors, including the size and characteristics of the hernia sac, the surgeon's experience and preference, and the patient's individual clinical presentation. The choice between excising or leaving the hernia sac should be made on a case-by-case basis, taking into account the specific circumstances of each patient and weighing the potential risks and benefits of each approach [9]. Further research and clinical studies are needed to provide clearer guidance on this aspect of CDH repair.

Minimally invasive techniques, such as laparoscopy, thoracoscopy and robotic, have been used successfully for the repair of some types of CDH. Laparoscopic repair is a technique used for MH repair, offering advantages such as shorter recovery time and reduced postoperative pain compared to open surgery. The first laparoscopic repair of a congenital MH in a child is credited to Georgacopulo et al., who performed the procedure in 1992 [10]. This landmark operation marked a significant advancement in the surgical treatment of MH, demonstrating the feasibility and benefits of a minimally invasive approach in pediatric patients. Thoracoscopic repair may be preferred for certain types of diaphragmatic hernias that are more difficult to access from the abdomen, such as Bochdalek diaphragmatic hernias [4, 8].

Robotic repair for congenital diaphragmatic hernias is similar to laparoscopic repair, but it confers several advantages over traditional laparoscopic surgery. The robotic arms provide a greater range of motion and enhanced precision, allowing for more intricate repairs and finer suturing. This may lead to better surgical outcomes and a reduction in the risk of complications. Additionally, the high-definition 3D imaging system provides a clearer view of the surgical field, allowing for more accurate placement of instruments and reducing the risk of complications. Robotic surgery allows for ergonomic positioning of the surgeon at a console away from the operating table, minimizing physical strain and fatigue during prolonged procedures. This can contribute to improved surgeon comfort and concentration, potentially leading to better surgical outcomes [11, 12].

However, robotic surgery isn’t appropriate for all patients, and it may not be widely available in all hospitals or medical centers. Robotic surgical systems are expensive to acquire and maintain, requiring significant investment in equipment, training, and maintenance. Robotic surgery requires specialized training and expertise, and the initial learning curve can lead to longer operative times and potentially higher rates of complications during the early stages of adoption. Furthermore, it heavily relies on sophisticated technology and equipment, making it vulnerable to technical malfunctions, system failures, or software glitches. In the event of a technical issue, the surgical procedure may need to be interrupted or converted to an alternative approach, potentially increasing the risk of adverse outcomes [11, 12].

The decision to use robotic-assisted surgery should be based on careful consideration of the specific clinical circumstances, surgeon expertise, and available resources.

## Conclusions

The atypical clinical presentations seen in cases of late-presenting MH can significantly impact the diagnosis of this condition. Patients who develop symptoms later in life generally have a favorable prognosis, as associated complications like pulmonary hypoplasia and pulmonary hypertension tend to be milder or even absent. However, it is crucial to maintain a high level of suspicion in every child exhibiting chronic, irremediable respiratory issues and gastrointestinal disturbances, as late-presenting MH can pose a life-threatening risk. Timely diagnosis and appropriate treatment are paramount for achieving positive outcomes. Laparoscopic repair represents a feasible option for addressing anterior diaphragmatic defects and may be considered as the primary approach for treating MH in suitable candidates. This method is both easily applicable and safe, offering favorable outcomes when properly executed.
